# Impurity Profiling of Dinotefuran by High Resolution Mass Spectrometry and SIRIUS Tool

**DOI:** 10.3390/molecules27165251

**Published:** 2022-08-17

**Authors:** Xianjiang Li, Wen Ma, Bingxin Yang, Mengling Tu, Qinghe Zhang, Hongmei Li

**Affiliations:** 1 Key Laboratory of Chemical Metrology and Applications on Nutrition and Health for State Market Regulation, Division of Metrology in Chemistry, National Institute of Metrology, Beijing 100029, China; 2 State Key Laboratory of Natural and Biomimetic Drugs, School of Pharmaceutical Sciences, Peking University, Beijing 100191, China; 3 Beijing Advanced Innovation Center for Soft Matter Science and Engineering, State Key Laboratory of Organic-Inorganic Composites, College of Chemical Engineering, Beijing University of Chemical Technology, Beijing 100029, China

**Keywords:** fragment tree, impurity profiling, SIRIUS, dinotefuran

## Abstract

Dinotefuran (DNT) is a neonicotinoid insecticide widely used in pest control. Identification of structurally related impurities is indispensable during material purification and pesticide registration and certified reference material development, and therefore needs to be carefully characterized. In this study, a combined strategy with liquid chromatography high-resolution mass spectrometry and SIRIUS has been developed to elucidate impurities from DNT material. MS and MS/MS spectra were used to score the impurity candidates by isotope score and fragment tree in the computer assisted tool, SIRIUS. DNT, the main component, worked as an anchor for formula identification and impurity structure elucidation. With this strategy, two by-product impurities and one stereoisomer were identified. Their fragmentation pathways were concluded, and the mechanism for impurity formation was also proposed. This result showed a successful application for combined human intelligence and machine learning, in the identification of pesticide impurities.

## 1. Introduction

Neonicotinoids (NEOs), chemically similar to nicotine, are the most widely used insecticides in the global market [1]. They interfere with the nicotinic acetylcholine receptor of the pest’s nervous system. Based on the mode of action classification scheme, they are classified in group 4A by the Insecticide Resistance Action Committee [2]. As the first member of the third-generation NEOs (furanicotinyl class), dinotefuran (DNT) has no chloropyridine or chlorothiazole ring. DNT is a chiral pesticide and was commercialized by Mitsui Chemicals in 2002 [3]. It is a systemic insecticide and has no cross-resistance with former NEOs. As the DNT-related patents have expired, new registrations from different suppliers are increasing. During active substance evaluation and pesticide registration, sufficient impurity identification is indispensable, because the safety of a pesticide not only relies on the active substance, but also on the contained impurities.

Due to its widespread usage, DNT has been one of the most frequently reported insecticides found in food and environmental samples, including oranges [4], berries [5], Chinese cabbage [6] and environmental water [7,8]. Additionally, the detection rate of DNT was 100% in 52 urine samples from Japanese adults [9] and 96% in 200 serum samples from South China [10]. According to the JMPR report, DNT had a reproductive and developmental toxicity in mammals, and the estimated acute reference dose was 1 mg/kg [11]. With such wide and persistent contamination, DNT is raising more and more concern. Recently, China issued the new national food safety standard (GB 2763-2021), in which DNT was strictly restricted in 52 foodstuffs. In the European Union, DNT has not been approved for use in plant protection products. It is obvious that the accurate determination of DNT is of great importance. To address this problem, certified reference materials (CRMs) are widely used to guarantee accurate results [12,13]. During DNT CRM development, impurity identification and quantification are significant for purity value assignment [14]. The impurities are usually process-related and degradation compounds. To the best of our knowledge, there is no impurity profile reported for high-purity DNT material.

Impurity identification and structure elucidation by mass spectrometry is highly challenging work. High-resolution mass spectrometry (HRMS) has the advantages of high resolution, mass accuracy and sensitivity [15,16]. Liquid chromatography (LC) coupled to HRMS is an important technique for analyzing untargeted impurities [17,18]. Orbitrap is a powerful mass analyzer with a high acquisition speed, resolving power, mass accuracy, and sensitivity [19]. Therefore, it has great potential for purity identification. SIRIUS is the best-of-class computer-assisted tool for metabolite formula annotation [20]. With this tool, isotope pattern analysis is used for MS data to find molecular formulae, and fragmentation tree is used for MS/MS data to elucidate chemical structure. Algorithms including Bayesian analysis and Maximum A Posteriori estimation work to score structure candidates [21]. The annotation is database-independent, and it also integrates CSI:FingerID to search structure databases [22].

In this work, a new strategy combining LC-HRMS and SIRIUS was developed for the identification of structurally related impurities in DNT material. HRMS was adopted to acquire the MS and MS/MS spectra of impurities. Isotope score and fragment tree in SIRIUS were used to score the impurity candidates with algorithms. DNT, the main component, worked as an anchor for structure elucidation and impurity confirmation. Finally, two impurities and one stereoisomer were observed and comprehensively characterized. This combined strategy improved identification accuracy and speed. To the best of our knowledge, this is the first DNT impurity profile that uses combined human intelligence and machine learning, providing a new perspective and instructive method for impurity identification in pesticides.

## 2. Materials and Methods

### 2.1. Chemicals and Materials

DNT (CAS: 165252-70-0) was from Toronto Research Chemicals (Nanjing, China). Methanol was bought from Merck (Darmstadt, Germany). Stock solutions were prepared at 1 mg/mL in methanol. Deionized water was obtained from a Milli-Q plus apparatus (Milford, MA, USA). All of the above chemicals were used directly without any further purification.

### 2.2. Instruments

LC-UV was performed using a Shimadzu LC-20A with 20AT binary pump and M20A PDA detector (Kyoto, Japan). Three different columns from Agilent were compared: the ZORBAX Eclipse plus C_18_ column, Eclipse plus C_8_ column and Eclipse XDB-CN column (250 × 4.6 mm, 5 μm). LC-HRMS was performed using a Thermo Fisher Vanquish Q-Exactive Plus system, which had a quaternary pump and an orbitrap mass analyzer (San Jose, CA, USA). An XP205 balance from Mettler Toledo (Greifensee, Switzerland) was used to weigh compounds.

### 2.3. Impurities Separation

The content of structural-related impurities were closely related to the DNT production method. The impurities mainly derive from residual, intermediate, side reactions or degradation products, which usually have a similar chemical structure. DNT may be produced with several different precursors, such as tetrahydro-3-furanmethanamine and N,O-dimethyl-N’-nitroisourea [23], 3-(methanesulfonyloxymethyl)tetrahydrofuran and 1,5-dimethyl-2-nitroimino-hexahydro-1,3,5-triazine, amongst others [3]. Impurity separation was carried out using an LC-UV instrument. Considering their polarity and hydrophobicity, three widely used Agilent columns were compared, including the Eclipse XDB CN, Eclipse plus C_8_ and Eclipse plus C_18_. The comparison was carried out with an isocratic mobile condition of 20% methanol. DNT stock solution (5 μL) was injected without dilution. The flow rate was set to 1.0 mL/min and the whole column temperature was kept at 30 °C. The LC conditions were optimized to separate DNT and impurities, including column and elution program. The online UV spectra were recorded in the wavelength range from 200 to 400 nm. The content of DNT was calculated with the ratio between its peak area and the total area of all detected peaks under the same wavelength. Since the peak area of different impurities depended strongly upon the detected wavelength, an uncertainty from wavelength was introduced with the quantification method, together with the repeatability of random effect.

### 2.4. Impurities Identification

The DNT and impurities were analyzed with the LC-HRMS system. The LC condition was the same as for the former LC-UV method. Due to the high concentration of DNT, its eluent was cut into waste using a valve switch. The electrospray ion source was in positive mode as follows: spray voltage, 3 kV; aux gas heater temperature, 320 °C; capillary temperature, 320 °C; auxiliary gas pressure, 15 arbitrary units; sheath gas pressure, 45 arbitrary units. Full scan and parallel reaction monitoring (PRM) modes were used for the isotope pattern analysis of precursor ions and the fragmentation trees of product ions, respectively. For full scan mode, the resolution was 70,000 with an AGC target of 1e^6^. During PRM experiments, data were collected at the resolution of 70,000 with an AGC target of 1e^5^. Quadrupole was operated as the first mass analyzer and the orbitrap as the second mass analyzer. The normalized collision energy (NCE) was optimized according to the analyte. Mass spectra were obtained in centroid mode over the range *m*/*z* 100–800. To guarantee mass accuracy, the orbitrap spectrometer was calibrated routinely, as required. The data acquisition and processing were performed by Thermo TraceFinder^®^ and Freestyle^®^ software.

### 2.5. Impurities Elucidation

The MS and MS/MS data of DNT and impurities were exported by Freestyle^®^, and containing only the *m*/*z* value and signal intensity. MS data were identified through isotope pattern analysis in SIRIUS. The molecular formula was selected using the isotope and Zodiac scores [24] and human judgment. The calculated molecular formula was compared with DNT to find the difference moiety. The MS/MS data were further identified through fragmentation trees in SIRIUS. Meanwhile, the MS/MS spectrum was compared with DNT to find the same or different fragment ions. The chemical structure of impurities were selected using the tree score and human judgment. Moreover, the CSI:FingerID helped to elucidate the fragment mechanism. 

## 3. Results and Discussion

### 3.1. Impurities Separation

For structural analog impurity analysis, one challenge was the baseline separation of DNT from each impurity. As shown in Figure S1, DNT and impurities had a relatively weaker retention on the Eclipse XDB CN, because it was a polar column. In the chromatogram from Eclipse plus C_18_, the peak in DNT was quite tailing. Better separation was realized on the Eclipse plus C_8_ column. Therefore the Eclipse plus C_8_ column was selected for further mobile phase optimization. This type of column was also chosen in the CIPAC method 749 for DNT analysis [25].

**Figure 1:**
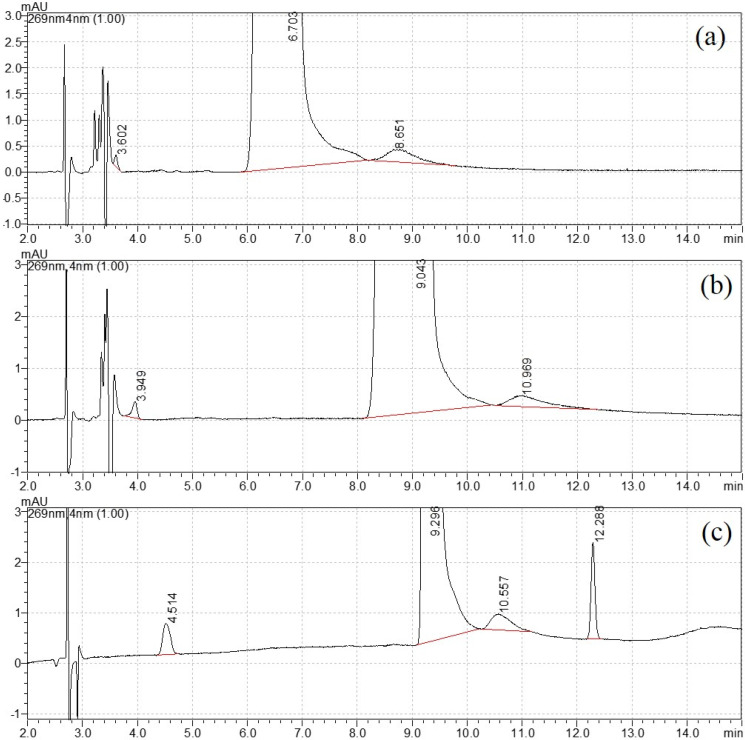
Mobile phase optimization: (**a**) 20% methanol, (**b**) 15% methanol and (**c**) gradient elution.

The mobile phase elution program was then optimized with the variation in water and methanol. For the isocratic elution of 20% methanol, the separation was not satisfactory, as shown in Figure 1a. When the percentage of methanol reduced to 15%, a new peak appeared close to the void time in Figure 1b. Therefore, gradient elution was selected for better separation. After careful optimization, the final elution program started with 10% methanol and increased to 50% at 10 min, then returned to the initial state at 11 min and equilibrated for the next injection. The retention time for four peaks was 4.499 min, 9.280 min, 10.554 min and 12.286 min, respectively (Figure 1c). The width of peak 3 was close to peak 2 (DNT), and this uncommon broadening needed further attention. Their UV spectra (220–300 nm) are shown in Figure S2. Similar spectra were observed with strong adsorption peaks appearing between 250 and 290 nm, with no adsorption peak above 300 nm. This UV profile confirmed that they had similar chromophores.

### 3.2. Impurities Identification

To avoid contamination, DNT peak was cut into waste between 6.5 and 7.3 min in the LC-HRMS system. Table 1 summarizes the accurate masses of the precursor and three product ions of DNT, as well as two impurities, respectively. The errors between the measured and calculated values ranged from −0.3 to 1.1 mDa (−5.2 to 4.6 ppm) with an average of 0.5 mDa, indicating that all the calculated elemental compositions were reliable. 

The spectra of DNT are shown in Figure S3 with [M+H]^+^ at 203.1136 and [M+Na]^+^ at 225.0954. Additionally, non-covalent dimer ions were observed with [2M+H]^+^ at 405.2199 and [2M+Na]^+^ at 427.2018. The dimer was probably formed through multi-hydrogen bonds over a proton bridge between two DNT [26]. When an NCE setting of 20 was applied, the precursor ion produced many fragment ions by eliminating NO_2_ and breaking the furan ring, with an *m*/*z* at 129.0898, 114.1029 and 87.0796. For peak 1 in Figure S4, [M+H]^+^ was at 133.0720, with [M+Na]^+^ at 155.0538. Non-covalent dimer ions were also observed, with [2M+H]^+^ at 265.1350 and [2M+Na]^+^ at 287.1168. When the NCE was 10, the precursor ion produced three prominent fragment ions at *m*/*z* 87.0796, 71.0611 and 58.0534. For peak 3 in Figure S5, [M+H]^+^ and [M+Na]^+^ spectra were the same as for DNT, while the non-covalent dimer ions ([2M+H]^+^ and [2M+Na]^+^) were much lower than for DNT. When the NCE was set as 30 for the [2M+H]^+^ ion, the precursor ion produced a typical [M+H]^+^ ion and similar fragment ions with DNT. This peak may be from an isomer of DNT. For peak 4 in Figure S6, the [M+H]^+^ was at 273.1554, with [M+Na]^+^ at 295.1373. Non-covalent dimer ions were also observed, with [2M+H]^+^ at 545.3010 and [2M+Na]^+^ at 567.2826. When the NCE was 25, the precursor ion produced a series of fragment ions at *m*/*z* 199.1321, 184.1445 and 156.1137.

From the above-acquired fragment ions and calculated neutral losses, some hints were useful for structure elucidation. Sodium adducts and non-covalent dimer ions were observed for all peaks. The DNT and other peaks were very similar in fragment ions. The ion (*m*/*z* 87.0796) appeared in all analytes. 

### 3.3. Impurities Elucidation

The spectra of impurities were further analyzed by SIRIUS (Version 4.9.15; Sebastian Böcker; Jena, Germany.) [27]. According to fragment trees, the calculated formulas and mass errors were summarized, as shown in Table 1. 

DNT was also analyzed as an unknown compound. A detailed result for DNT is shown in Figure S7. C_7_H_14_N_4_O_3_ ranked top in the calculated precursor ion. The SIRIUS score was 100.000% with an isotope score of 3.678 and a tree score of 69.368. The CSI:FingerID result had a score of −21.997 and 100% similarity with DNT. The Zodiac score was perfect, with 100.000%. All the data matched perfectly. Additionally, the fragmentation pathway and MS/MS spectra agreed with the published work [28].

For peak 1 in Figure S8, C_3_H_8_N_4_O_2_ was the only result. The SIRIUS and Zodiac score was perfect, with an isotope score of 3.580 and a tree score of 28.032. Compared with DNT, impurity 1 was less with C_4_H_6_O (tetrahydrofuran moiety). The CSI:FingerID result had a score of −78.876 and 56.757% similarity with N,N-dimethyl-N’-nitroguanidine (CAS 5465-97-4), which was a byproduct from the precursor. Its chemical structure and fragment ions are shown in Figure S4. A methyl isomer (CAS 101250-97-9), which ranked fourth, also had a high probability, with a slightly lower CSI:FingerID score and similarity. This tool works for an untargeted analysis in de novo annotation, so the number of candidate molecular formulae is enormous and molecular formulae are sometimes wrongly assigned. Further verification against a standard was necessary.

Peak 3 may be an isomer of DNT. According to the FAO specification of DNT [29], it actually consisted of E and Z isomers at the nitroguanidine moiety and an optical isomerism at the furan moiety. The stationary phase of the C_8_ column had no chirality, so this peak came from a stereoisomer. Interchange of the E and Z isomer occurred at room temperature; therefore, the peak broadened. The isomer had a different configuration, which affected the dimer formation. The IUPAC common name refers to the racemate and includes both the E and Z isomers. Finally, the peak was not considered as an impurity. 

For peak 4 in Figure S9, C_11_H_20_N_4_O_4_ was the best candidate, with SIRIUS and Zodiac scores of 100.000%, an isotope score of 5.357 and a tree score of 82.372. Compared with the DNT formula, there were more C_4_H_6_O elements (tetrahydrofuran moiety). This impurity may be from an over-reaction during DNT production. The CAS number of this impurity was 946009-58-1.

**Figure 2:**
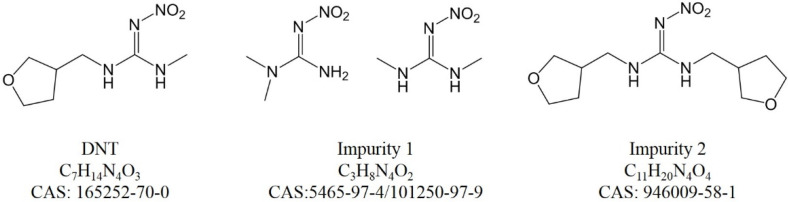
Chemical structures of DNT and impurities.

The chemical structures of DNT and the impurities are shown in Figure 2. Their hydrophobicity agreed with the LC elution order. It is possible that the impurities were different among various suppliers; the four characterized impurities would provide much help for future exploration.

### 3.4. Purity and Uncertainty Evaluation

Because these impurities had similar chemical structures to DNT, relative quantitation was carried out by the normalization of the peak area in the chromatogram. The maximum adsorption wavelengths were 267 nm, 268 nm, 270 nm and 269 nm for the four peaks. Their similar UV spectra came from the same nitroguanidine chromophore, and the tetrahydrofuran moiety had no obvious effect in this wavelength range. Therefore, 269 nm was selected for quantification. The calculated relative impurity content comprised 0.086% of the total chromatographic peak area, which indicated an extremely high purity of DNT material. In this context, detection response factors may be different between the impurity and DNT under the given experimental conditions. Uncertainty from the wavelength was introduced, along with the quantification method, according to the standard JJF 1855–2020 [30]. The chromatographic purity was 999.14 mg/g, with a standard uncertainty of 0.02 mg/g, covering the contribution from a difference in detection response factor and the repeatability of random effect.

## 4. Conclusions

In this study, a method based on LC-HRMS was developed for the comprehensive characterization of structurally related impurities in DNT material. Prior to the identification of the inherent impurities, the LC condition was carefully optimized to achieve good separation for the DNT isomer and two impurities. Subsequently, the separated impurities were clearly identified using LC-HRMS and elucidated by SIRIUS. One impurity was a DNT precursor and the other was from over-reaction. This work combined human intelligence with machine learning in impurity profiling analysis to simplify the tedious annotation process and to increase the elucidation accuracy, which helps greatly in pesticide registration and CRM development.

## Figures and Tables

**Table 1:** Exact masses of DNT and impurities.

Analytes	Assignment	Formular	Measured (*m*/*z*)	Calculated (*m*/*z*)	Error (mDa)	Error (ppm)
DNT	[M+H]^+^	C_7_H_14_N_4_O_3_	203.1136	203.1144	0.8	3.9
	[M+Na]^+^		225.0954	225.0964	1.0	4.4
	[M-74]^+^	C_5_H_10_N_3_O	129.0898	129.0902	0.4	3.3
	[M-89]^+^	C_5_H_11_N_3_	114.1029	114.1031	0.2	1.9
	[M-116]^+^	C_3_H_8_N_3_	87.0796	87.0797	0.1	0.6
Peak 1	[M+H]^+^	C_3_H_8_N_4_O_2_	133.0720	133.0725	0.5	3.8
	[M+Na]^+^		155.0538	155.0545	0.7	4.6
	[M-46]^+^	C_3_H_8_N_3_	87.0796	87.0797	0.1	0.6
	[M-62]^+^	C_3_H_6_N_2_	71.0611	71.0609	−0.2	−2.5
	[M-75]^+^	C_2_H_5_N_2_	58.0534	58.0531	−0.3	−5.2
Peak 4	[M+H]^+^	C_11_H_20_N_4_O_4_	273.1554	273.1563	0.9	3.3
	[M+Na]^+^		295.1373	295.1383	1.0	3.2
	[M-74]^+^	C_9_H_16_N_3_O_2_	199.1316	199.1321	0.5	2.5
	[M-89]^+^	C_9_H_17_N_3_O	184.1445	184.1450	0.5	2.7
	[M-117]^+^	C_7_H_13_N_3_O	156.1132	156.1137	0.5	3.1

## Data Availability

Not applicable.

## Supplementary Materials

The following supporting information can be downloaded at: https://www.mdpi.com/article/10.3390/molecules27165251/s1. Figure S1: Column selection for DNT (a) Eclips XDB CN, (b) Eclips plus C_8_, (c) Eclips plus C_18_. Figure S2: UV spectrum of impurities and DNT. Figure S3: Mass spectra of DNT: (a) MS and (b) MS/MS. Figure S4: Mass spectra of impurity 1: (a) MS and (b) MS/MS. Figure S5: Mass spectra of peak 3: (a) MS and (b) MS/MS. Figure S6: Mass spectra of impurity 2: (a) MS and (b) MS/MS. Figure S7: SIRIUS result for DNT. Figure S8: SIRIUS result for impurity 1. Figure S9: SIRIUS result for impurity 2.
