# Supplementary material for: Impurity Profiling of Dinotefuran by High Resolution Mass Spectrometry and SIRIUS Tool
Source: Molecules. 2022 Aug 17;27(16):5251. doi: 10.3390/molecules27165251 (PMC9415319; doi:10.3390/molecules27165251)
Supplement: Supplementary file 1 [file molecules-27-05251-s001.zip › molecules-1848700-supplementary.pptx]

## Slide 1
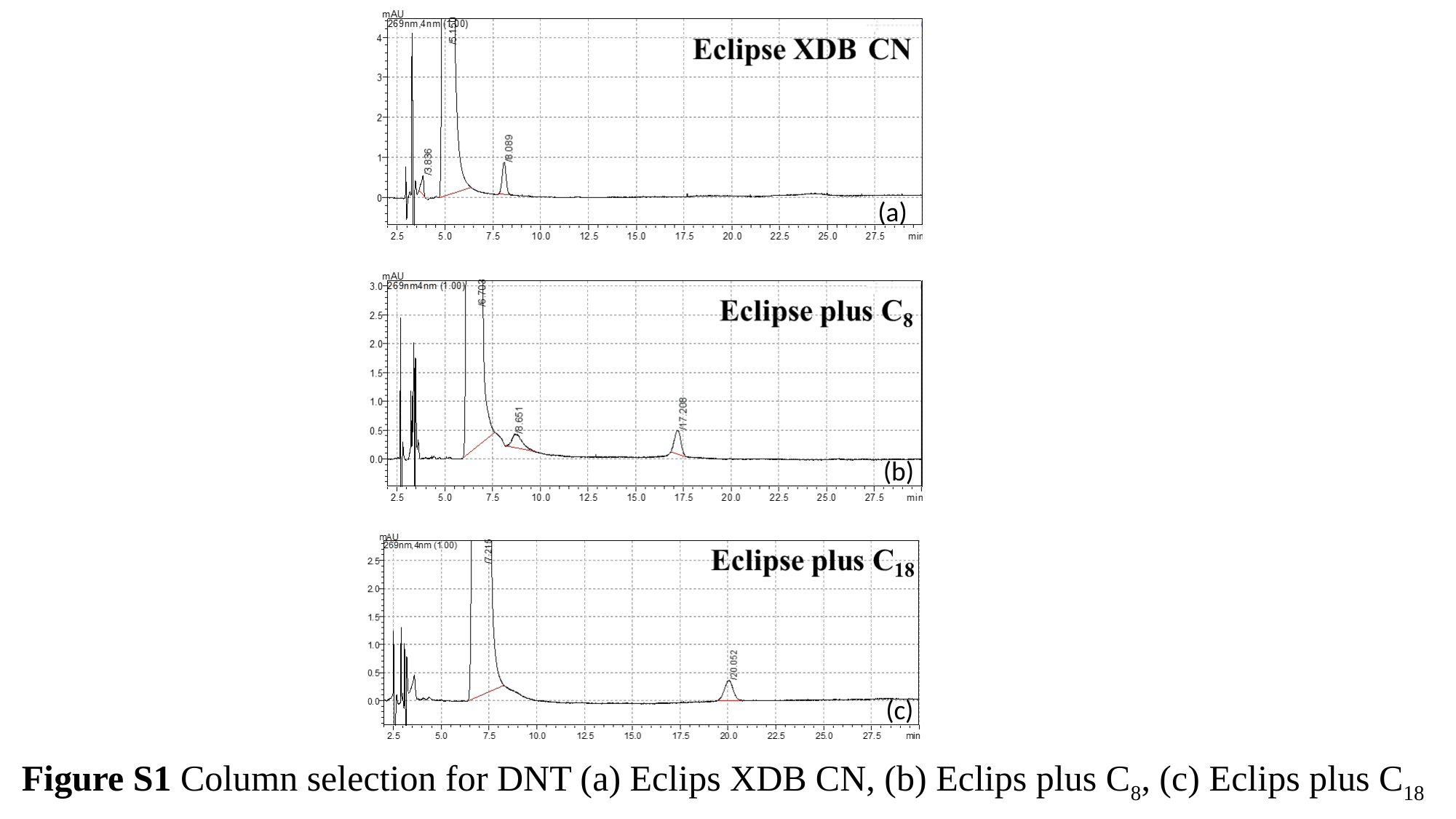

(a)
(b)
(c)
Figure S1 Column selection for DNT (a) Eclips XDB CN, (b) Eclips plus C8, (c) Eclips plus C18

## Slide 2
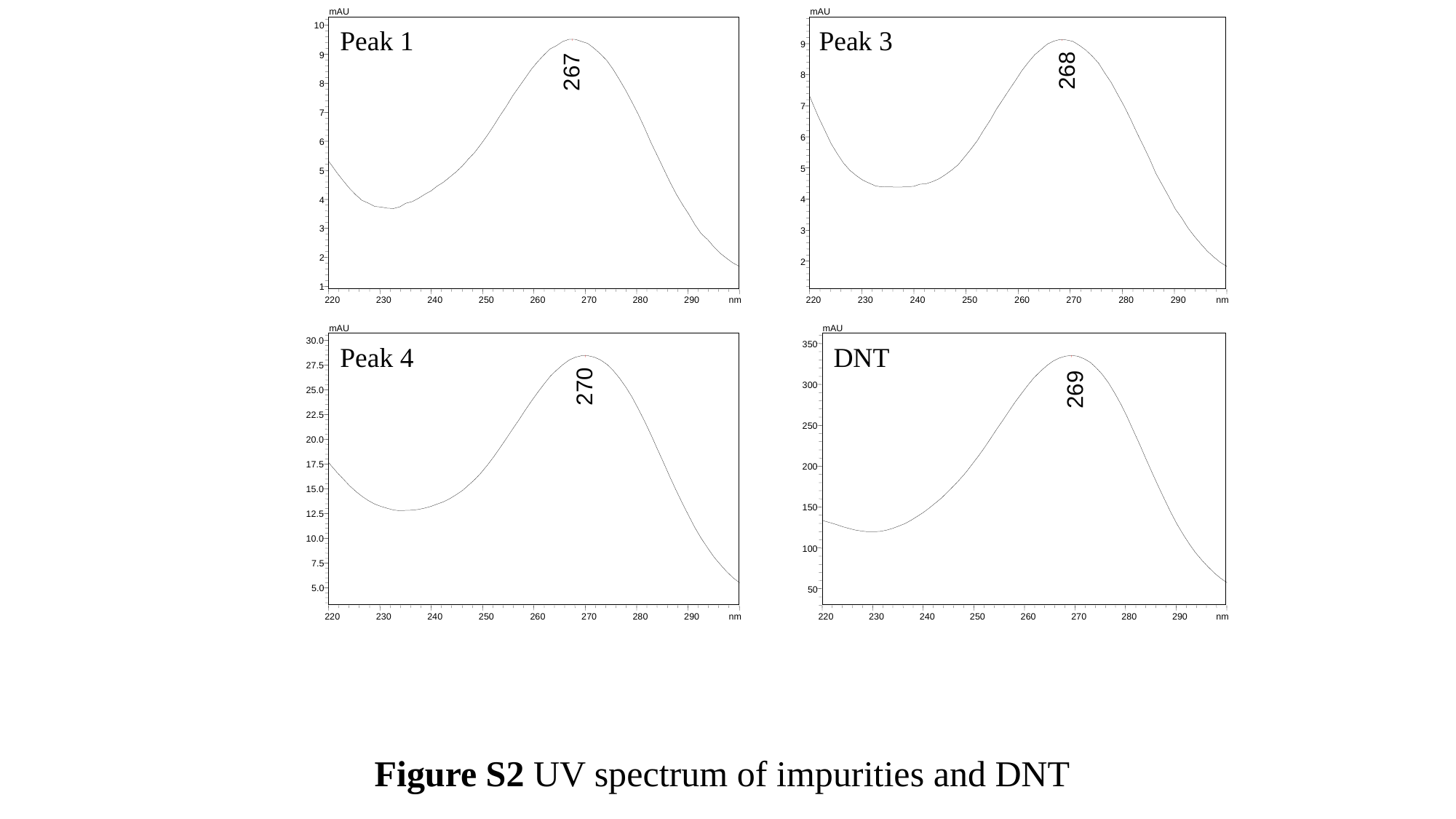

mAU
10
9
267
8
7
6
5
4
3
2
1
220
230
240
250
260
270
280
290
nm
mAU
9
268
8
7
6
5
4
3
2
220
230
240
250
260
270
280
290
nm
Peak 1
Peak 3
mAU
30.0
27.5
270
25.0
22.5
20.0
17.5
15.0
12.5
10.0
7.5
5.0
220
230
240
250
260
270
280
290
nm
mAU
350
269
300
250
200
150
100
50
220
230
240
250
260
270
280
290
nm
Peak 4
DNT
Figure S2 UV spectrum of impurities and DNT

## Slide 3
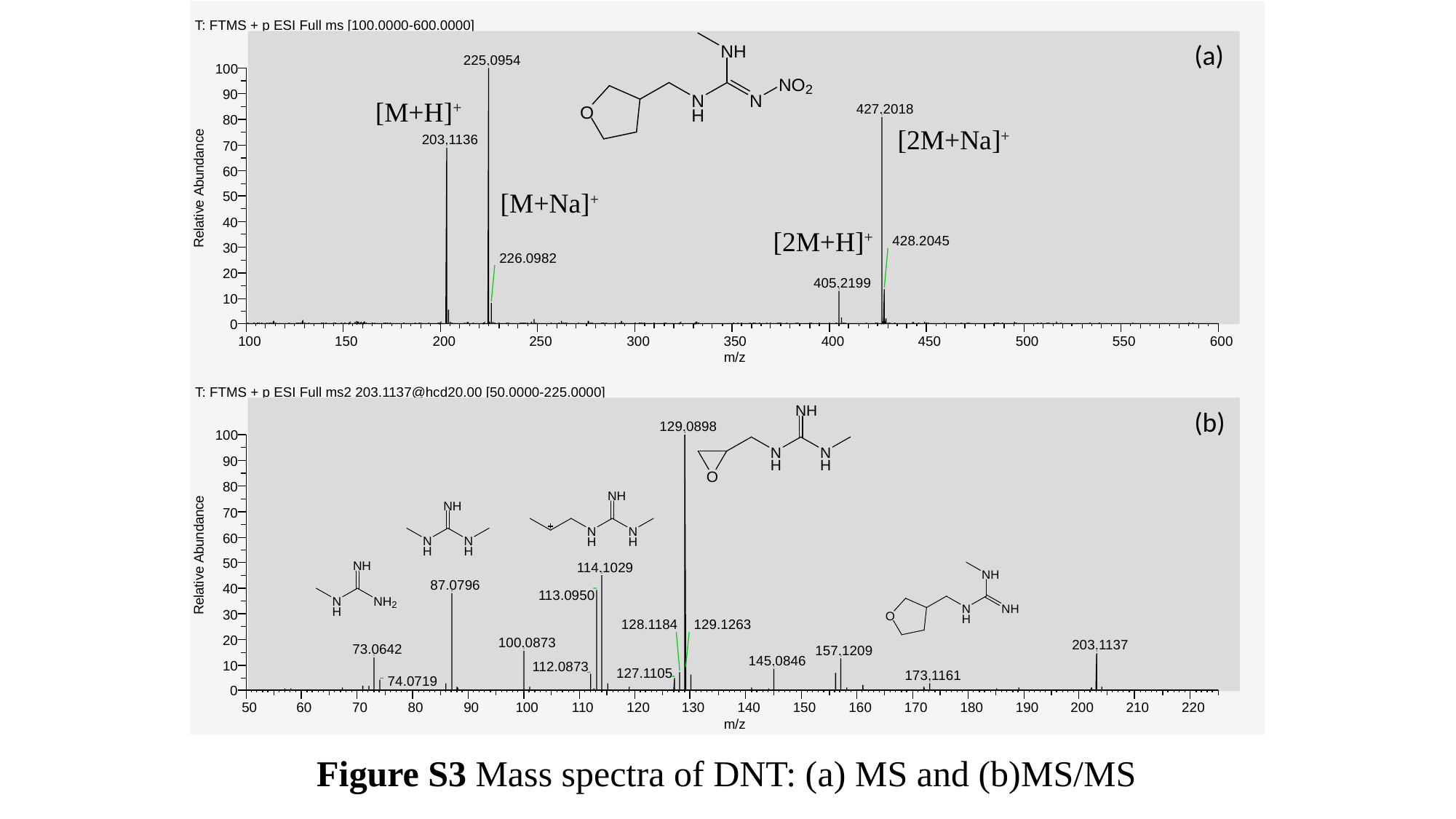

T: FTMS + p ESI Full ms [100.0000-600.0000]
225.0954
100
90
427.2018
80
e
c
203.1136
70
n
a
d
n
60
u
b
A
50
e
v
i
t
40
a
l
e
428.2045
R
30
226.0982
20
405.2199
10
0
100
150
200
250
300
350
400
450
500
550
600
m/z
(a)
[M+H]+
[2M+Na]+
[M+Na]+
[2M+H]+
T: FTMS + p ESI Full ms2 203.1137@hcd20.00 [50.0000-225.0000]
129.0898
100
90
80
e
c
70
n
a
d
n
60
u
b
A
50
114.1029
e
v
i
t
87.0796
40
a
113.0950
l
e
R
30
128.1184
129.1263
20
100.0873
203.1137
73.0642
157.1209
145.0846
10
112.0873
127.1105
173.1161
74.0719
0
50
60
70
80
90
100
110
120
130
140
150
160
170
180
190
200
210
220
m/z
(b)
Figure S3 Mass spectra of DNT: (a) MS and (b)MS/MS

## Slide 4
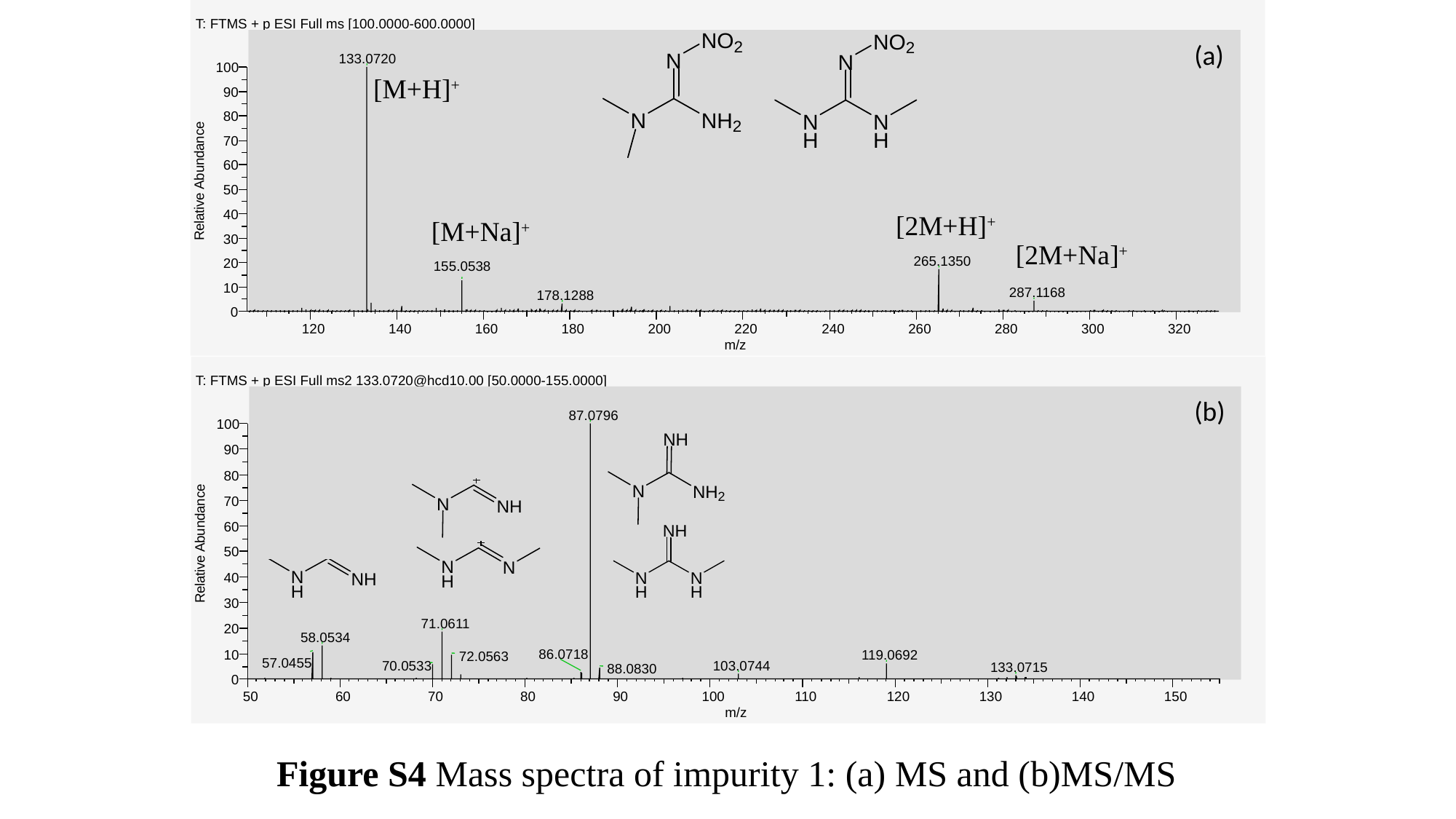

T: FTMS + p ESI Full ms [100.0000-600.0000]
133.0720
100
90
80
e
c
n
70
a
d
n
60
u
b
A
50
e
v
i
t
40
a
l
e
R
30
265.1350
20
155.0538
10
287.1168
178.1288
0
120
140
160
180
200
220
240
260
280
300
320
m/z
(a)
[M+H]+
[2M+H]+
[M+Na]+
[2M+Na]+
T: FTMS + p ESI Full ms2 133.0720@hcd10.00 [50.0000-155.0000]
87.0796
100
90
80
e
c
70
n
a
d
n
60
u
b
A
50
e
v
i
t
40
a
l
e
R
30
71.0611
20
58.0534
86.0718
10
119.0692
72.0563
57.0455
70.0533
103.0744
133.0715
88.0830
0
50
60
70
80
90
100
110
120
130
140
150
m/z
(b)
Figure S4 Mass spectra of impurity 1: (a) MS and (b)MS/MS

## Slide 5
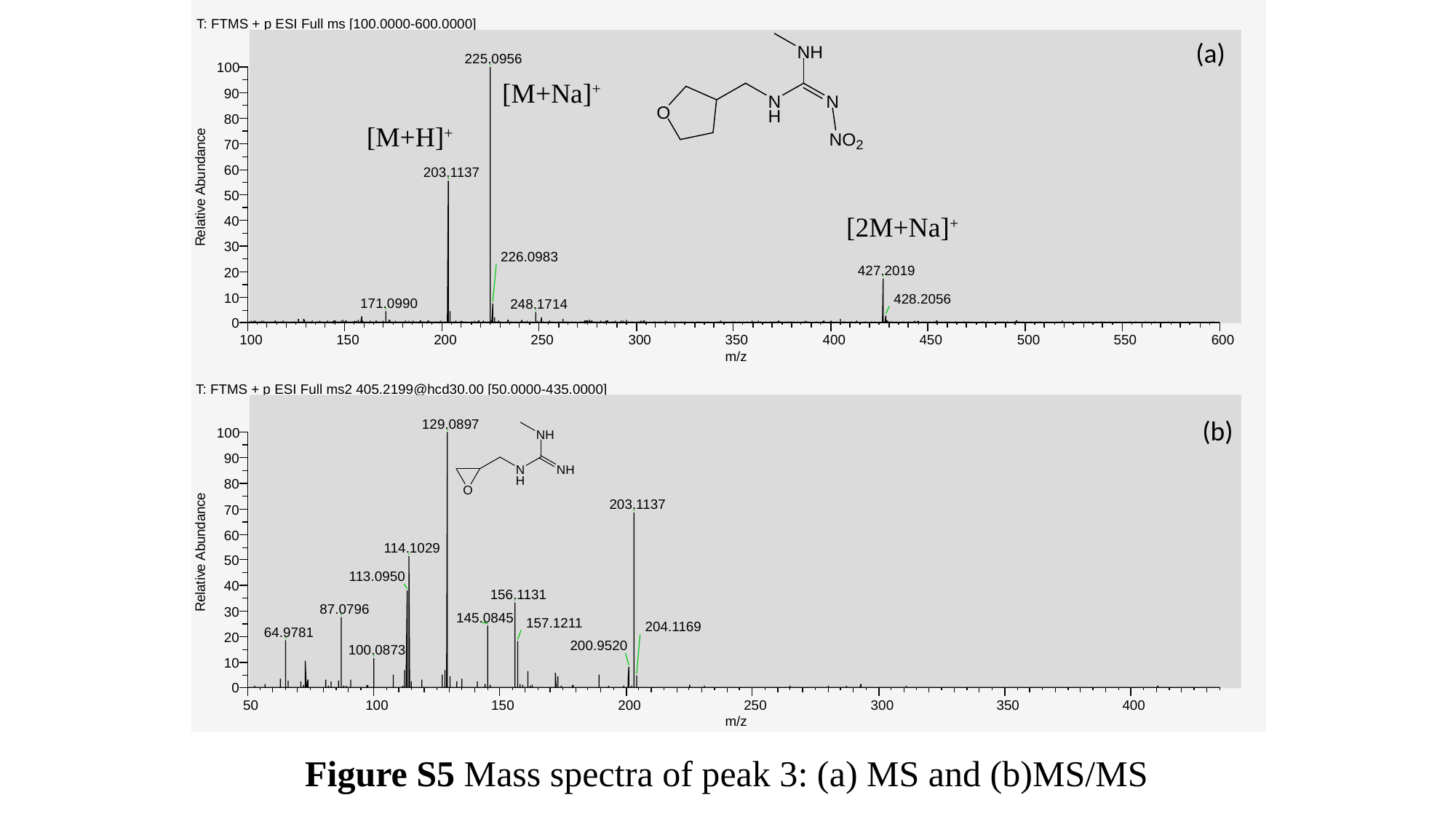

T: FTMS + p ESI Full ms [100.0000-600.0000]
225.0956
100
90
80
e
c
70
n
a
d
n
60
203.1137
u
b
A
50
e
v
i
t
40
a
l
e
R
30
226.0983
427.2019
20
10
428.2056
171.0990
248.1714
0
100
150
200
250
300
350
400
450
500
550
600
m/z
(a)
[M+Na]+
[M+H]+
[2M+Na]+
T: FTMS + p ESI Full ms2 405.2199@hcd30.00 [50.0000-435.0000]
129.0897
100
90
80
e
c
203.1137
70
n
a
d
n
60
u
b
114.1029
A
50
e
v
113.0950
i
t
40
a
l
156.1131
e
R
87.0796
30
145.0845
157.1211
204.1169
64.9781
20
200.9520
100.0873
10
0
50
100
150
200
250
300
350
400
m/z
(b)
Figure S5 Mass spectra of peak 3: (a) MS and (b)MS/MS

## Slide 6
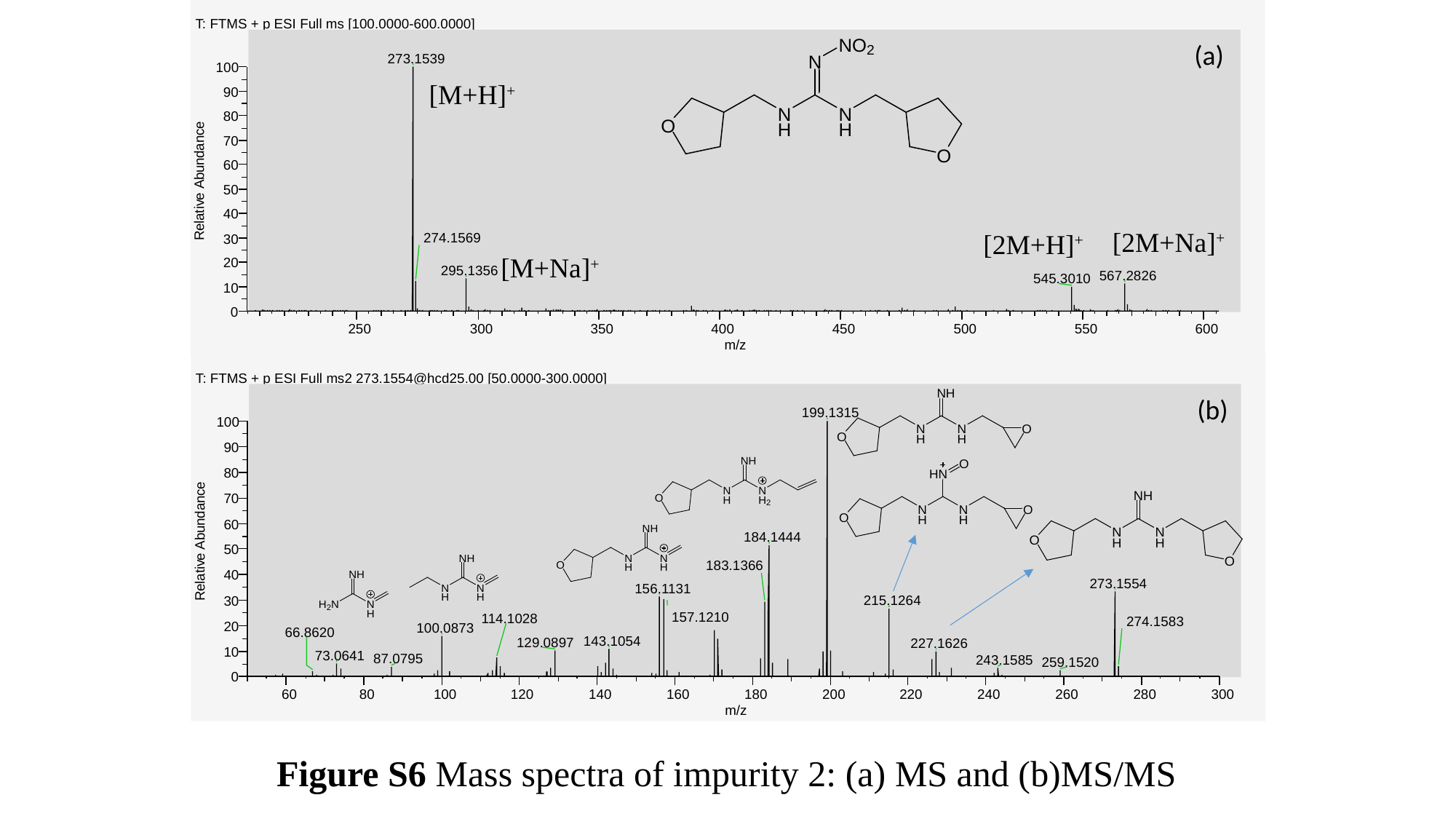

T: FTMS + p ESI Full ms [100.0000-600.0000]
273.1539
100
90
80
e
c
n
70
a
d
n
60
u
b
A
50
e
v
i
t
40
a
l
e
R
274.1569
30
20
295.1356
567.2826
545.3010
10
0
250
300
350
400
450
500
550
600
m/z
(a)
[M+H]+
[2M+Na]+
[2M+H]+
[M+Na]+
T: FTMS + p ESI Full ms2 273.1554@hcd25.00 [50.0000-300.0000]
199.1315
100
90
80
e
c
70
n
a
d
n
60
u
b
184.1444
A
50
e
v
183.1366
i
t
40
a
l
273.1554
e
156.1131
R
215.1264
30
157.1210
114.1028
274.1583
20
100.0873
66.8620
143.1054
129.0897
227.1626
10
73.0641
87.0795
243.1585
259.1520
0
60
80
100
120
140
160
180
200
220
240
260
280
300
m/z
(b)
Figure S6 Mass spectra of impurity 2: (a) MS and (b)MS/MS

## Slide 7
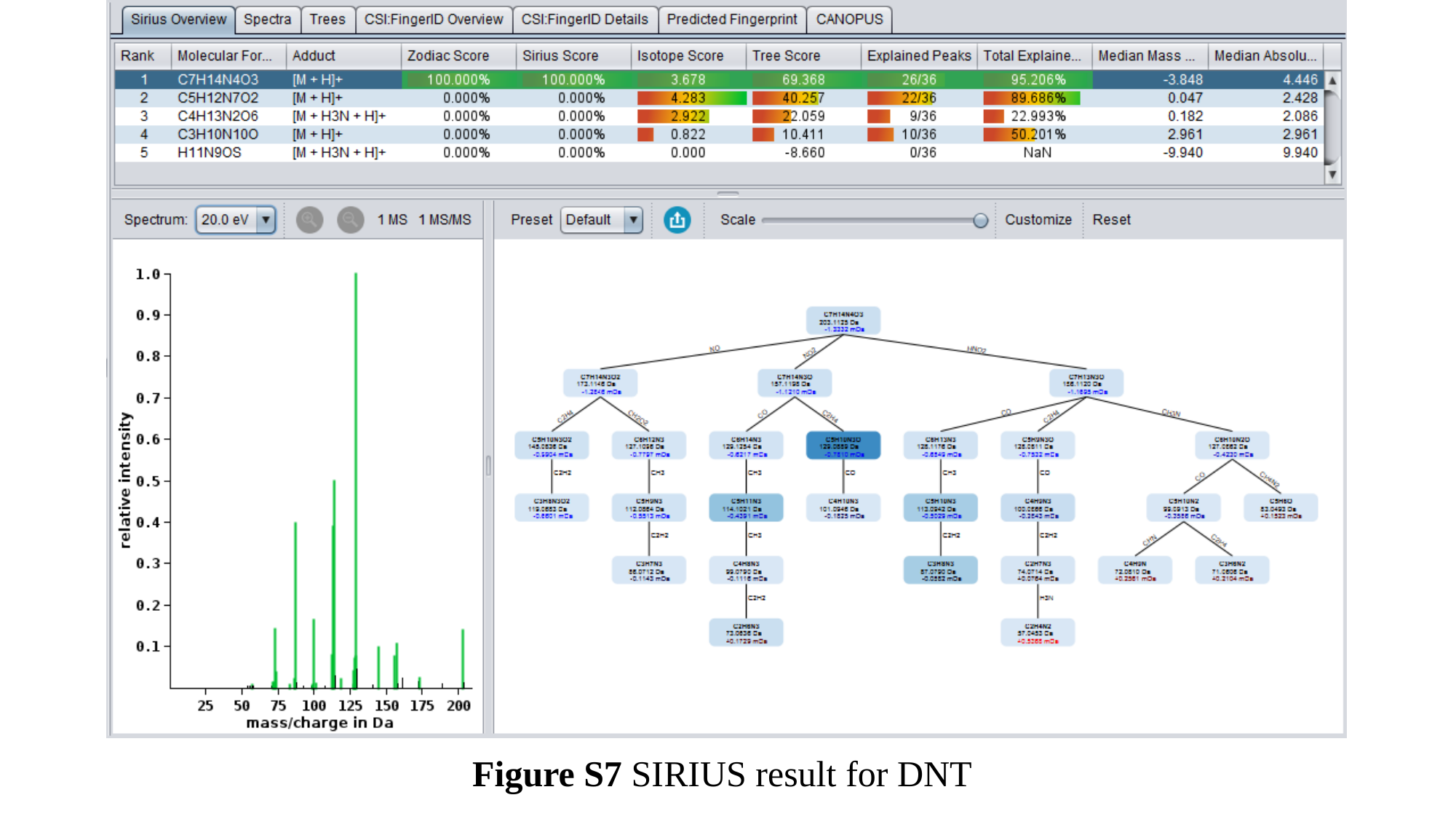

Figure S7 SIRIUS result for DNT

## Slide 8
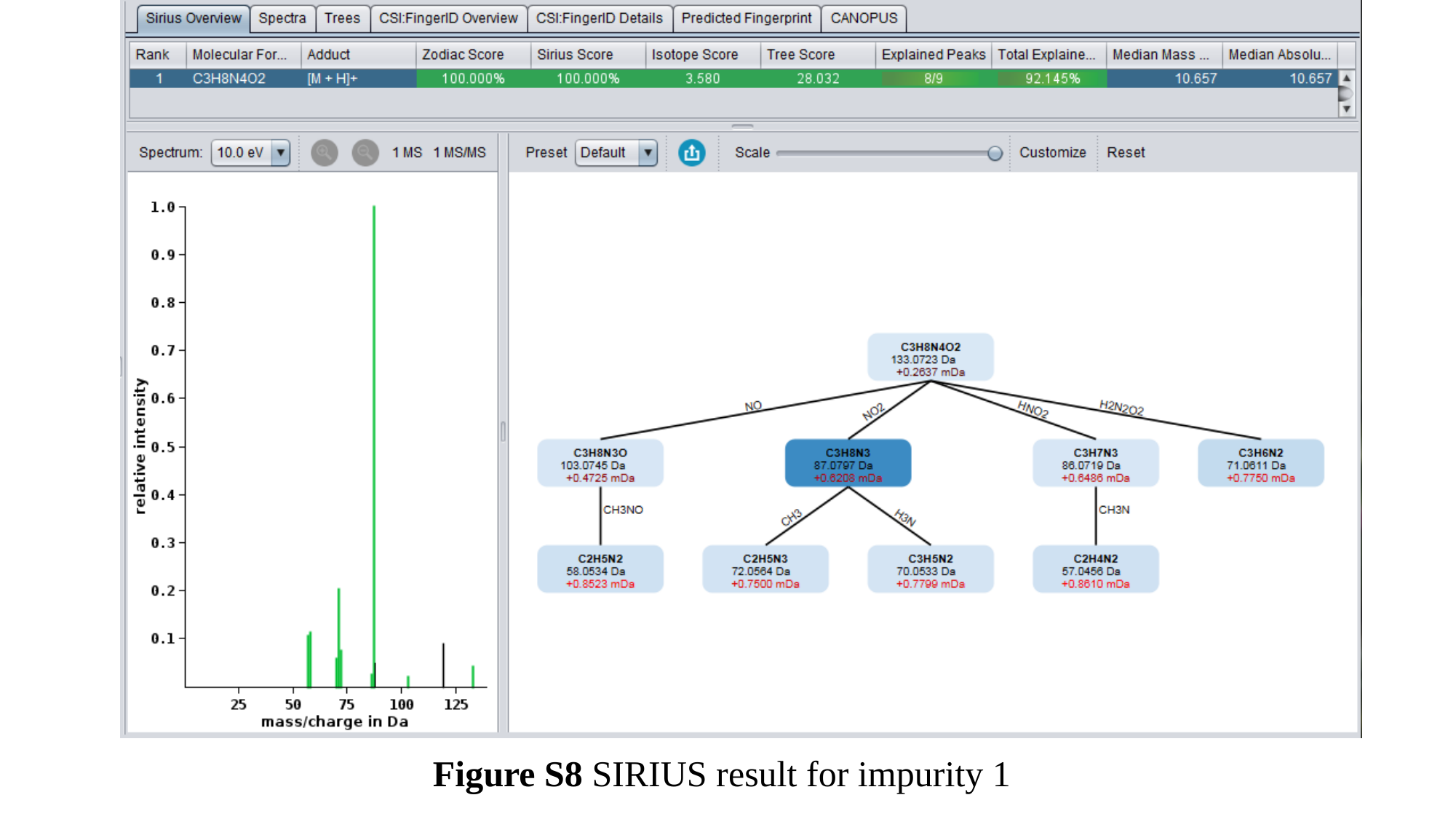

Figure S8 SIRIUS result for impurity 1

## Slide 9
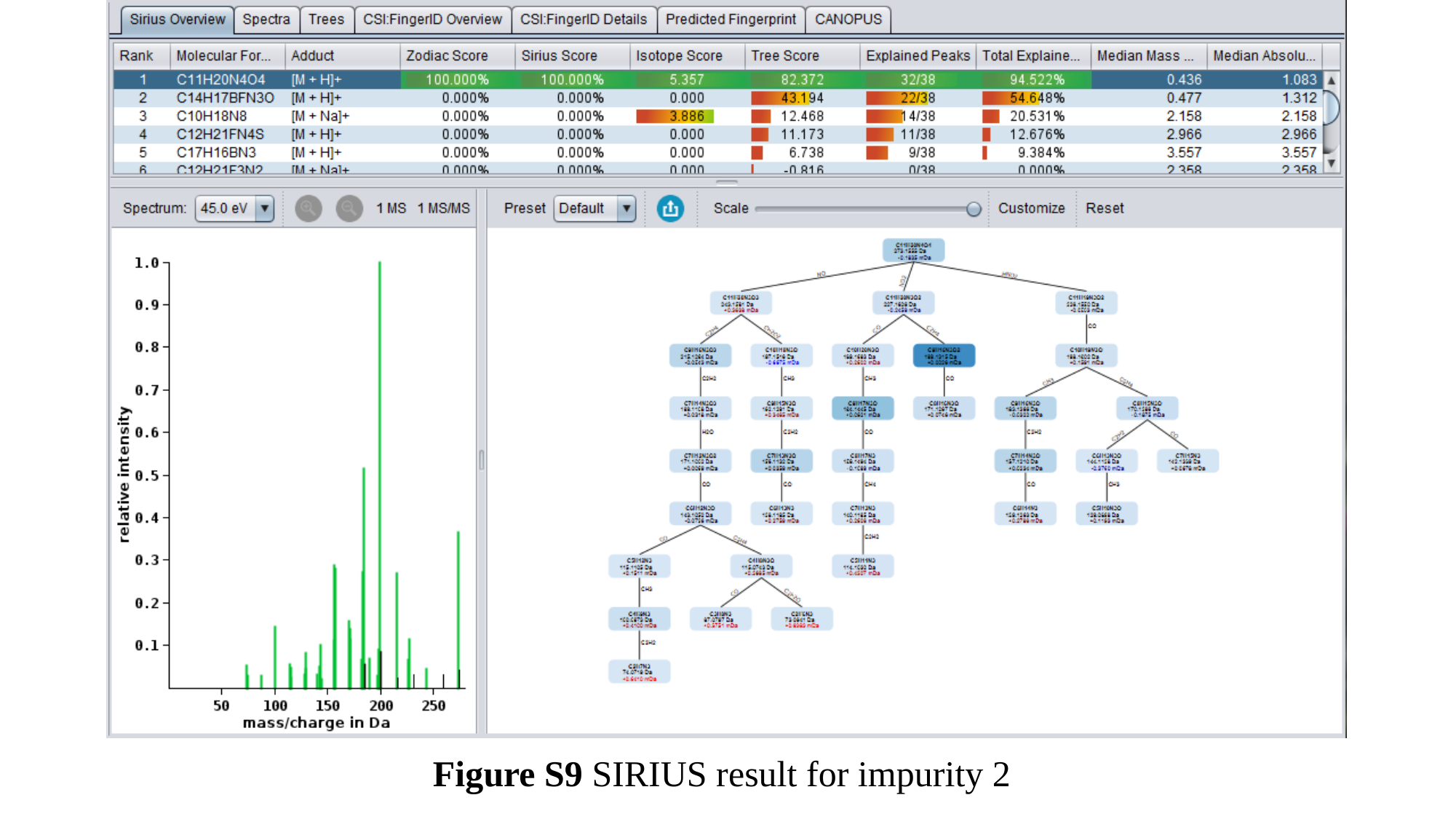

Figure S9 SIRIUS result for impurity 2
